# Reliability of the Output Sports Inertial Measurement Unit in Measuring a Reactive Strength Index from the Drop Jump and 10-5 Rebound Jump Test

**DOI:** 10.3390/sports14010015

**Published:** 2026-01-04

**Authors:** Conor P. Clancy, Kieran D. Collins, Thomas M. Comyns

**Affiliations:** 1 Department of Physical Education and Sport Sciences, University of Limerick, V94 T9PX Limerick, Ireland; tom.comyns@ul.ie; 2 School of Biological Health and Sport Sciences, Technological University Dublin, Tallaght Campus, D24 FKT9 Dublin, Ireland; kieran.collins@tudublin.ie; 3 Gaelic Sport Research Centre, Technological University Dublin, Tallaght Campus, D24 FKT9 Dublin, Ireland; 4 Sport and Human Performance Research Centre, University of Limerick, V94 T9PX Limerick, Ireland; 5 Health Research Institute, University of Limerick, V94 T9PX Limerick, Ireland

**Keywords:** stretch shortening cycle, plyometrics, performance testing, strength and conditioning, inertial measurement unit

## Abstract

This study examined the trial-to-trial reliability and usefulness of the Output Sports inertial measurement unit (IMU) in measuring reactive strength index (RSI) derived from the Drop Jump (DJ) and 10-5 rebound jump test (10-5 RJT). Twenty-three male elite intercounty hurlers (mean ± SD; age: 24.3 ± 3.7 years, mass: 88.0 ± 6.3 kg, height: 183.8 ± 5.8 cm, experience at elite level: 5.8 ± 3.8 years) performed three trials each of the DJ and 10-5 RJT, on familiarisation and testing days. There was one week between familiarisation and testing. Reliability was determined by intraclass correlation (ICC) and coefficient of variation (CV) analyses. Usefulness was assessed by comparing typical error (TE) with the smallest worthwhile change (SWC). Both the DJ and 10-5 RJT were reliable in determining RSI, with CV ≤ 10% and ICC ≥ 0.8. The TE was 0.09 and 0.08 for the DJ and 10-5 RJT, respectively. Both tests were unable to detect the SWC, rating them as ‘marginal’; however, they were rated as ‘good’ in detecting moderate change in RSI. The Output Sports IMU is reliable in determining RSI for the DJ and 10-5 RJT; however, it is unable to detect the SWC. Future research must determine validity of the Output Sports IMU in measuring RSI.

## 1. Introduction

The performance of athletes is influenced by various strength-based qualities, such as relative strength [1]. Reactive strength is described as the ability to change from an eccentric to a concentric contraction [2]. The coupling of eccentric and concentric muscle actions forms the stretch-shortening cycle (SSC) [3]. Stretch-shortening cycle actions can be described as fast or slow, with fast-SSC couplings demonstrated in ground contact times (GCT) of ≤0.25 s and slow-SSC couplings being >0.25 s [4]. The actions can be quantified through the use of the reactive strength index (RSI), which can be calculated as jump height (JH) divided by GCT and may be used as a measure of fast-SSC capabilities. Lower GCT and higher JH will result in a greater RSI score. Keeping the amortisation phase low, which is the phase between eccentric and concentric contractions, is the most consequential phase of a reactive jump when trying to reduce GCT and improve fast-SSC movements in sporting populations [5]. Plyometric exercises are an important element of a training programme for practitioners to aid the improvement of athletes’ SSC capabilities through force generation [6].

There are various reactive strength test protocols, which are used extensively in practical settings in the determination of RSI. Two such tests, which are utilised in optimising the force production capabilities in fast-SSC movements, are the Drop Jump (DJ) and the 10-5 rebound jump test (10-5 RJT). A DJ is performed when an athlete drops from a box or platform and upon hitting the ground, rebounds vertically as quickly and as powerfully as possible [7]. The reliability of the DJ in measuring RSI has been researched previously [8,9], with it demonstrating reliability within acceptable limits of coefficient of variation (CV) and intraclass correlation coefficient (ICC) [8,9]. Byrne et al. [8] tested the inter-day reliability of RSI and the optimal drop height through use of the Optojump™ and found adequate reliability of RSI, with an ICC value of 0.87 and CV of 4.2%. Markwick et al. [9] tested the intra-day reliability of RSI at varying heights for the Drop Jump through the use of a force plate, with each height also demonstrating acceptable reliability with an ICC > 0.8 and CV < 10%. The 10-5 RJT involves an athlete completing a countermovement jump followed by ten repeated pogo jumps [10]. These studies used different pieces of equipment for testing, and as such, it should be noted that the reliability within their results are equipment- and protocol-specific. Similarly to the DJ, minimising GCT and rebounding powerfully in a vertical direction with small angular displacements at the hip, knee, and ankle in the 10-5 RJT are important in demonstrating higher RSI values. Harper et al. [10] designed the 10-5 RJT protocol where only the five highest RSI values calculated from the ten pogo jumps are included for analyses. There has been limited research on the reliability of the 10-5 RJT in determining RSI; however, recent studies demonstrate that it has adequate inter-day and test–retest reliability [10,11]. Comyns et al. [11] examined the 10-5 RJT in relation to the inter-day reliability of RSI through the use of the Optojump™, and similar to the DJ, the CV of 9.8% and ICC of 0.89 for the full participant cohort fell within acceptable ranges. However, in detecting the smallest worthwhile change (SWC), its usefulness was questionable.

Reliability of test procedures are important for monitoring of sport performance and training effects [12]. It is pertinent that the equipment used in test procedures undergoes rigorous reliability testing to assess athlete performance correctly. If the collected and collated data is unreliable, this will then have an adverse effect on how the data is analysed [13]. Incorrect inference of unreliable data could have a negative impact on athlete monitoring and perceived performance indicators. The usefulness of testing equipment should also be scrutinised during reliability testing. The equipment used must be sensitive and be able to express meaningful changes over time in performance parameters [14]. Sensitivity in meaningful change is an important part of performance in sporting populations. It affords practitioners the opportunity to closely monitor training loads and to maximise performance through training prescription [11].

The use of electronic devices for testing for RSI within sport settings has become common in practice. There are various devices currently utilised by sports practitioners which can calculate RSI from SSC testing protocols. Force plates have long been established as the ‘gold standard’ in the measurement of RSI [15]. Devices such as Optojump™ have also demonstrated to be a valid measure in determining RSI [16]. Force plates and Optojump™ are relatively expensive and may not be of practical use to a coach due to budgetary constraints though. Jump mats are a relatively inexpensive device that are also employed by practitioners in SSC testing; however, the validity of these mats in determining RSI is questionable [17]. Kenny et al. [17] compared an electronic switch mat to an AMTI OR6-5 force platform for validity and reliability, and whilst it was valid in determining squat jump and countermovement jump height, it was unable to accurately measure GCT and JH in the DJ. As such, the DJ was not a valid measure for RSI through the use of the electronic switch mat. It may not be pertinent for practitioners to employ the use of a jump mat whilst using the DJ concurrently with similar RSI testing protocols such as a 10-5 RJT due to its demonstration of systematic bias in the DJ.

Other devices capable of measuring RSI are inertial measurement units (IMUs). IMUs offer practitioners and coaches an inexpensive, portable device with which to measure RSI activity in a timely manner [18]. IMUs are small wearable sensors that are composed of accelerometers, gyroscopes, and magnetometers, which can measure and interpret force-velocity data and angular limb movement [18]. IMUs such as accelerometers have been shown to have a strong correlation to ‘gold standard’ force plates [15]. Marković et al. [19] recently examined the accuracy of an IMU in estimating vertical jump height, demonstrating strong agreement with force plate measures. IMUs provide practitioners with the ability to navigate large testing groups in short periods of time due to their portability and ease of use. A commercially available IMU, the Push Band 2.0 has been shown to have excellent reliability in measuring RSI; however, the system demonstrated systematic bias when the validity of the unit was concurrently tested [20]. The Output Capture IMU from Output Sports is a relatively new IMU available to practitioners. The wearable sensor includes software with a user interface that analyses strength and performance-based testing protocols. The sensor can be used to test the DJ and 10-5 RJT and capture the resultant RSI data. The device employs machine learning algorithms to assess movements in jumps through accelerometer signals [21]. The collected information can be stored in Output’s cloud-based system, allowing for access to data.

The current availability of the literature on the concurrent reliability and validity of IMUs is sparse. To the knowledge of the authors, there is currently only one published piece of literature on the reliability of the Output Sports IMU in the measurement of RSI [22]. Montoro-Bombú et al. [22] examined the concurrent validity and reliability of the Output Sports Capture unit during a DJ. The study found that GCT was overestimated, and that JH and RSI were underestimated in relation to concurrent force plate data. Inter-measure reliability of RSI demonstrated an ICC of 0.772 and CV of 32.22%; however, the study did not evaluate a meaningful change between trials [22]. Emergent technologies such as IMUs are becoming increasingly used by practitioners for training monitoring and performance enhancement, so establishing their measurement reliability is critical to their practical implementation [23]. Therefore, the reliability of the Output IMU in determining RSI must be tested further and within the 10-5 testing protocol.

As the present investigation was methodological in nature, focusing on device reliability rather than sport-specific performance outcomes, one team of elite trained hurlers was recruited, as they were familiar with plyometric-based movements and capable of producing consistent maximal efforts during fast-SSC tasks. Hurling is a high intensity stick and ball field sport, where two teams execute offensive and defensive skills at high speeds, with the goal of the game being to outscore the opposing team across the course of the game [24].

The current study aimed to establish the trial-to-trial reliability and usefulness of the Output Sports IMU in assessing RSI derived from the DJ and the 10-5 RJT.

## 2. Materials and Methods

### 2.1. Experimental Approach to the Problem

The study was conducted using a cross-sectional design with repeated trials of the DJ and 10-5 RJT. Participants took part in two sessions: firstly, a familiarisation session, then a testing session. A standardised warm-up was conducted followed by three trials each of the DJ and 10-5 RJT in each session. Data was recorded using the Output Capture (Output Sports). Data collected from the testing day was used to determine the reliability and usefulness of the Output Capture in measuring RSI in the DJ and 10-5 RJT. The testing session followed the same protocol as the familiarisation session. There was a one-week period between sessions.

### 2.2. Participants

Twenty-three male (mean ± SD; age: 24.3 ± 3.7 years, mass: 88.0 ± 6.3 kg, height: 183.8 ± 5.8 cm, experience at elite level: 5.8 ± 3.8 years) field sport athletes, who played elite-level hurling for a senior intercounty team, took part in this study. Each participant had more than two years of resistance training experience and were familiar with bilateral hopping and fast-SSC exercises from previous training within the sport. Ethical approval was obtained from the institutional ethics committee prior to the commencement of the study. Before testing, participants signed informed consent documents and completed a physical activity readiness questionnaire (PAR-Q). No participant made a declaration in the PAR-Q that precluded them from participating in the study. The procedures of this study were conducted in accordance with the Declaration of Helsinki and received ethical approval from the research ethics committee of the University of Limerick (2016_10_04_EHS).

### 2.3. Procedures

Prior to both the familiarisation session and the testing session, the testing protocol was explained to each participant. Both sessions were conducted at the same time of day, one week apart to avoid the impact of circadian variation [25]. The DJ and 10-5 RJT were visually demonstrated to the participants by the practitioner to guide and improve their coordination pattern of the exercises [26]. The warm-up consisted of five minutes of low intensity on the spot jogging followed by lower body dynamic stretching. Inclusion of jogging in the warm-up prepares participants for impact in plyometric activity [6]. Furthermore, the implementation of dynamic stretching in the warm-up has been extensively shown to elicit greater increases in RSI in comparison to static stretching [27], as well as improving power [28], and overall jump performance when combined with a general warm-up activity such as jogging [29]. The dynamic stretches performed were bodyweight squats, reverse lunges, and lateral lunges. Each dynamic stretch was performed for ten repetitions. Participants also performed ten repetitions each of high knees and pogo jumps, which simulated the plyometric elements of the exercises to be performed in testing. Two minutes rest was provided after the warm-up before the commencement of the DJ and 10-5 RJT trials.

**Figure 1:**
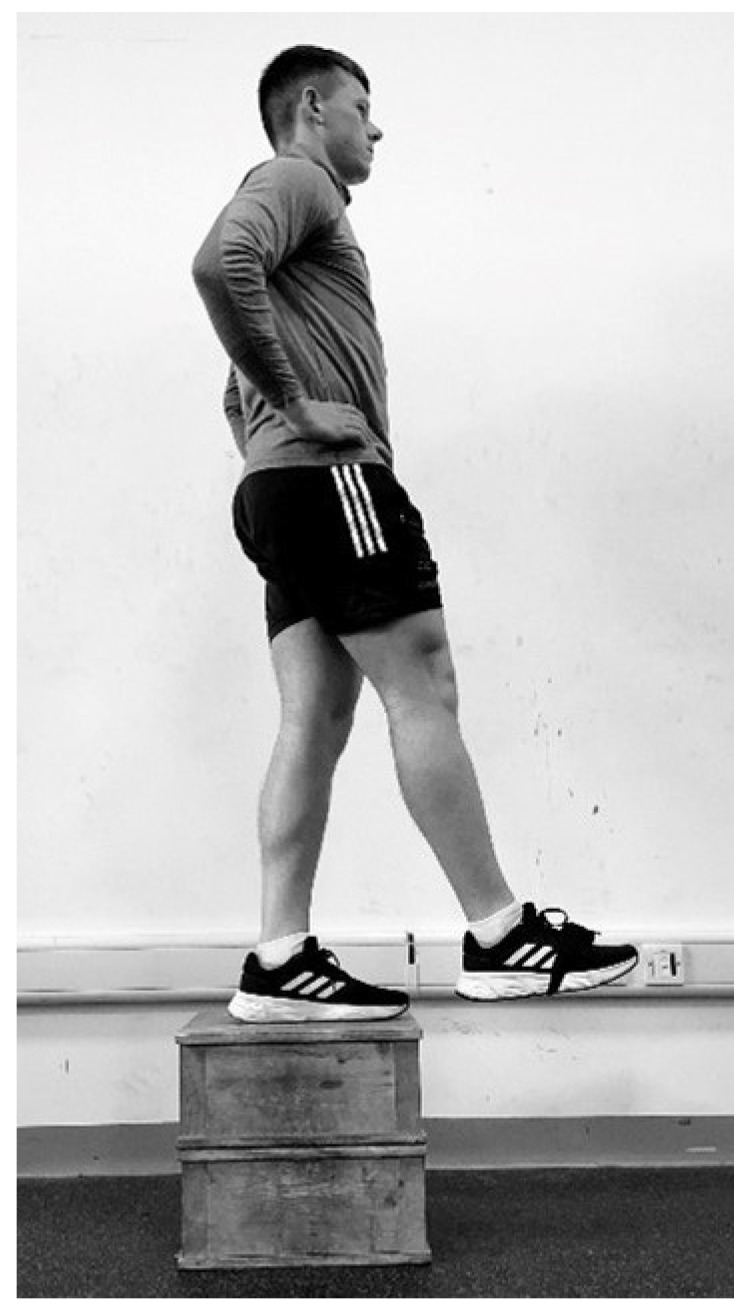
Example of Output IMU placement within foot strap for the Drop Jump.

Three trials of the DJ were completed first followed by three trials of the 10-5 RJT. The height of the box for the DJ was set at 30 cm, which is similar to previous research protocols [15,17,30]. A rest period of at least 1:5 was employed between repetitions to limit induced fatigue between trials [6]. The rest period between repetitions was one minute, with a rest period of two minutes between the DJ and the 10-5 RJT. Coaching applications were similar for both tests due to the similarity of the movements. Participants were instructed to jump and land in the same spot throughout testing [11]. Standardisation of hands on hips throughout was repeated to participants to limit arm and upper-body movement throughout the exercises [31]. The Output Capture IMU (V1) (Dimensions: 51 mm × 34 mm × 14 mm; Weight: 24 g) was used to calculate RSI in testing. The unit consists of a gyroscope, an accelerometer, and a magnetometer. It was operating at 512 Hz at the time of testing. Following completion of the warm-up, the unit was inserted in the foot strap provided by Output Sports and placed around the foot of the participant, facing upward on the front of the shoe (See Figure 1). The device was securely fastened with a Velcro strip to limit the risk of movement throughout testing. The IMU was connected via Bluetooth to the Output Sports android mobile application (v0.5.5). The placement of the sensor was on the right foot for all participants involved for the DJ and remained on the same foot for the 10-5 RJT also.

External cues were provided by the researcher throughout the course of testing. An external focus of attention has been shown to improve jump performance [32]. It has also been shown to improve RSI in fast-SSC exercises such as the DJ when compared to neutral conditions [33]. Cues such as ‘Jump through the roof’, ‘Stay stiff like a spring’, and ‘The floor is a hot surface’ were provided to the participants throughout both sessions [11]. These cues were provided during testing for both the DJ and 10-5 RJT. Emphasis on ‘The floor is a hot surface’ and ‘Jump through the roof’ were provided during the DJ. Reducing GCT and maintaining JH are most important to DJ performance as it will result in increased power and resultant increased RSI [34]. ‘Stay stiff like a spring’ was utilised more during the 10-5 RJT. An external focus of attention aids motor learning more effectively than an internal focus [35], as such, no biomechanical focused feedback cues were issued to the participants by the practitioner during testing. External cues remained consistent across both tests for all participants.

### 2.4. Statistical Analyses

Significance for the study was set at *p* ≤ 0.05. Data was normally distributed as assessed by the Shapiro–Wilk test (*p* > 0.05). Statistical mean and standard deviation were calculated for each trial across the tested cohort. The reliability of both tests was determined by calculating the CV and ICC. Acceptable values for reliability are a CV ≤ 10% and an ICC ≥ 0.8 [36]. The CV and ICC with 95% confidence intervals were calculated using a Microsoft Excel spreadsheet [37]. The usefulness of the tests was conducted by comparing the typical error (TE) to the smallest worthwhile change (SWC). The SWC is calculated by multiplying the between-subject SD by effect sizes. When multiplied by 0.2 (SWC0.2), a small effect was determined, and when multiplied by 0.5 (SWC0.5), an alternate moderate effect was assessed. If the TE was below the SWC, the test was rated as ‘good’, ‘ok’ if both were similar, and ‘marginal’ if the TE was higher than the SWC [38].

## 3. Results

Table 1 represents the mean and standard deviation of participants for all three trials completed for the DJ and 10-5 RJT. All twenty-three participants completed the DJ; however, the 10-5 RJT trials are only based on the results of twenty participants. The remaining three participants did not complete the 10-5 RJT protocols, as they chose not to take part in this section of testing.

Table 2 highlights results for ICC, CV%, TE, SWC 0.2, and SWC 0.5. The usefulness of both tests is also described in Table 2. Both the DJ and 10-5 RJT were unable to detect a ‘small’ worthwhile change in RSI, as the TE was above the SWC. Both were able to detect a moderate SWC and were rated as ‘good’ due to the TE being below the SWC in that case.

**Figure 2:**
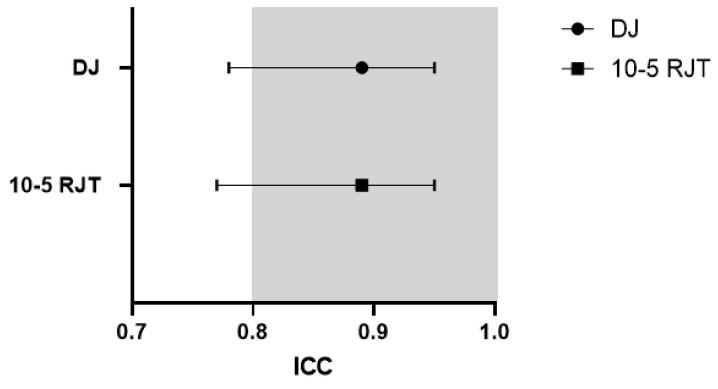
ICC ± 95% CI for DJ and 10-5 RJT jump protocols. Shaded region indicates area of acceptance of reliability. ICC—Intraclass Correlation Coefficient; DJ—Drop Jump; 10-5 RJT—10-5 Rebound Jump Test.

**Figure 3:**
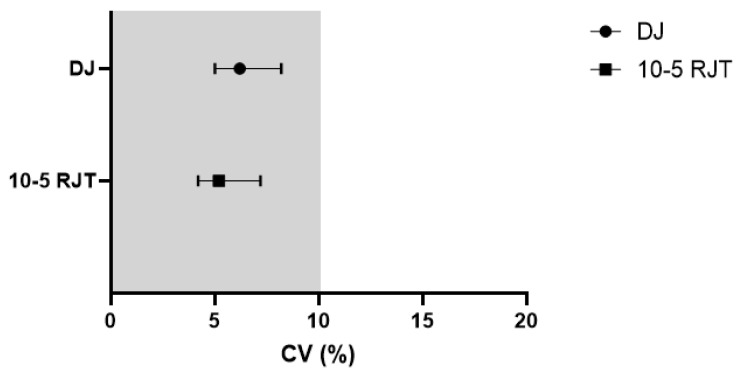
CV ± 95% CI for DJ and 10-5 RJT jump protocols. Shaded region indicates area of acceptance of reliability. CV—Coefficient of Variation; DJ—Drop Jump; 10-5 RJT—10-5 Rebound Jump Test.

**Figure 4:**
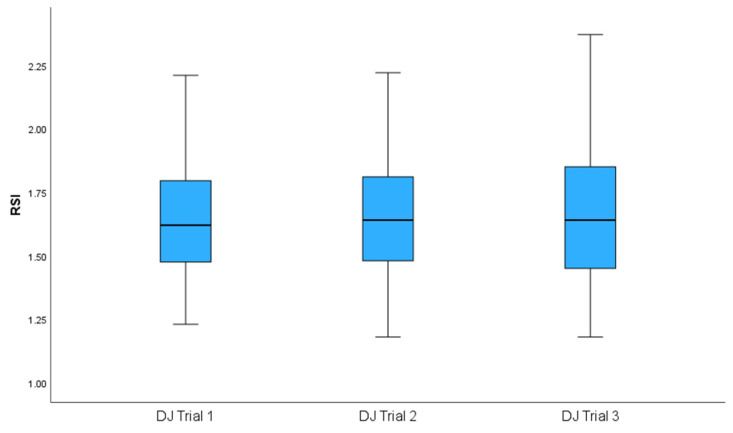
Boxplots showing the distribution of measurements across all participants for Trial 1, Trial 2, and Trial 3 for the Drop Jump. DJ—Drop Jump; RSI—Reactive Strength Index.

**Figure 5:**
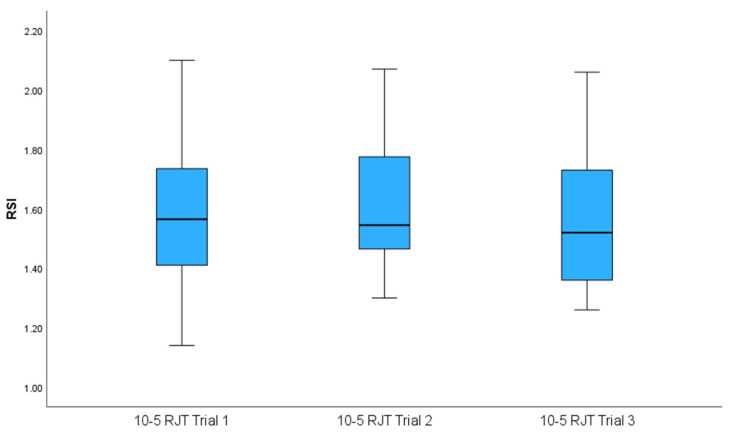
Boxplots showing the distribution of measurements across all participants for Trial 1, Trial 2, and Trial 3 for the 10-5 rebound jump test. 10-5 RJT—10-5 Rebound Jump Test; RSI—Reactive Strength Index.

Figure 2 and Figure 3 illustrate how both tests demonstrated acceptable reliability through ICC and CV. The means of both tests met the criteria of an ICC of ≥0.8 and a CV ≤ 10%. The shaded areas in both graphs indicate areas of acceptable reliability. Figure 4 and Figure 5 present boxplots of each trial for the DJ and 10-5 RJT, respectively. These boxplots show the distribution of measurements for all participants across Trials 1–3.

## 4. Discussion

The DJ and 10-5 RJT were deemed to be reliable in determining RSI values using the Output Capture IMU. The current study is the first to examine the reliability of the 10-5 RJT in measuring RSI through the use of the Output Capture IMU. Both the DJ and the 10-5 RJT satisfied the reliability criteria of an ICC ≥ 0.8 and a CV ≤ 10%. The findings of the current study throw questions over the ability of the Output device to identify the SWC of RSI in field-based athletes despite its relevant reliability in measuring it. The DJ had a TE of 0.09, with the SWC being 0.05. For the 10-5 RJT, the TE was 0.08 and the SWC 0.04. As the TE was greater than the SWC in both cases, the Output Capture unit was deemed unable to detect the SWC and was therefore rated as ‘marginal’. The Output device was able to determine a moderate change in RSI and was rated as ‘good’ due to the TE for both the DJ and 10-5 RJT being less than the SWC in this case. The results of the current study contrast with that of Montoro-Bombú et al. [22], whose ICC of 0.772 and CV of 32.22% for the DJ in measuring RSI, fall outside of the stated acceptable ranges.

The findings of this study on the Output Capture IMU in collecting RSI data from trial-to-trial will be of benefit to practitioners from a training and acute monitoring standpoint. Performance indicators may be an integral part of determining performance as agreed upon by a coach and athlete [39]. Fast-SSC exercises can be incorporated in programmes of field-based athletes in the training or maintenance of maximum sprint speed [40]. The usability of RSI in monitoring fast-SSC exercises such as the DJ and 10-5 RJT through devices such as an IMU are useful for practitioners when acutely monitoring such athletes. Therefore, it is imperative that coaches or practitioners are aware of the monitoring tools at their disposal and its subsequent reliability in the delivery of data. It is pertinent to note, though, that the DJ and 10-5 RJT should not be used interchangeably over time when monitoring RSI [41]. Furthermore, it is important that practitioners have TE and SWC data available when determining what device will be used in data capturing for RSI and general training practices. The Output device was unable to detect the SWC for the DJ and 10-5 RJT, which would have to be considered when monitoring field-based athletes for data analysis. Monitoring of training loads needs to be sensitive to small changes for the practitioner to monitor acute loading and athlete preparedness [11]. Beattie and Flanagan determined that when the SWC cannot be detected, sensitivity change in RSI could be determined using the CV in group analysis, and for an individual athlete, the change could be based on the individual’s CV over time [31]. Similarly in this study, a practitioner could use the CV to detect changes in performance over time with field-based athletes or could use the SWC to detect moderate changes in RSI through use of the Output Capture IMU.

Whilst the Output Capture unit was unable to detect the SWC of both the DJ and 10-5 RJT, it should be noted that the inability to detect the SWC may not solely be based on the data capturing abilities of the IMU but also on the jump test protocols. Previous studies found inability in detecting the SWC in both the DJ [31] and the 10-5 RJT [11,42]. Sensor placement may have influenced the reliability of IMU measurement. Different placement sites can affect vertical jump metrics, suggesting that small variations in attachment location could contribute to measurement noise and affect the device’s ability to detect meaningful changes in RSI [19]. Consistent placement and secure attachment of an IMU may minimise measurement noise and note more meaningful change over time [19]. Furthermore, differences in the usefulness of the tests could also be population-specific [11]. The current cohort demonstrated CVs of 6.2% and 5.2% for the DJ and 10-5 RJT, respectively. Markwick et al. [9] examined the reliability of the DJ in measuring RSI from trial-to-trial, with the cohort examined demonstrating CV results of 2.1% to 3.1% for DJ heights of 20–50 cm. The participants in that study were professional basketball players in comparison to elite amateur field sport athletes in the current study. The average RSI value of 2.1 in Markwick et al. [9] for the DJ was also higher than in the current study. The professional basketball players may have had more fast-SSC training than the current amateur cohort, which may explain some of the differences in CV. In addition to athlete population differences, variance in results could also be attributed to age-related factors. Doyle et al. [42] examined the trial-to-trial reliability of RSI derived from the 10-5 RJT in under seventeen, under nineteen, and senior female international soccer players. The results from Doyle et al. [42] showed CVs for RSI of 7.28%, 4.95%, and 3.15% in under seventeen, under nineteen, and senior players, respectively. The results obtained from the current study may indicate that the nature of the population group evaluated may have had an impact on the ability of the Output IMU in detecting meaningful change from trial-to-trial.

In addition, practitioners should be aware of the learning effect that comes with repeated trials and how this may have affected the TE of the Output Capture unit. It can be expected that with more familiarisation trials for each individual, the TE would reduce over a period of time [11]. There is also evidence based around how familiarisation of an exercise impacts the determinants of reliability in testing protocols [43]. Research into the isometric mid-thigh pull found that reliability increased across four familiarisation sessions which then provided actionable data that could be applied to monitoring [43]. The limited familiarisation trials in the current study potentially may have influenced the testing trials, whereby some of the participants may have still been in a state of learning of the prescribed movements by the end of the testing protocol. Furthermore, the limited research on the Output Capture IMU meant that only comparisons of the DJ in measuring RSI could be examined in relation to the current study. Whilst the data collected in the current study demonstrated adequate trial-to-trial reliability, there is currently no published validity research on the ability of the Output Capture IMU to accurately measure RSI in the 10-5 RJT. Therefore, it is not known whether the data that is being produced by the Output Capture IMU with regard to RSI derived from the 10-5 RJT is valid for testing within field based athletic populations.

The future research directions of the Output Capture IMU in measuring RSI are two-fold. Firstly, the reliability of the IMU to measure RSI in both the DJ and 10-5 RJT needs to be researched extensively with larger cohorts. The reliability and usefulness of the Output Capture IMU needs to be tested on an inter-session basis, as previous studies have examined intra-session variability only for the DJ [22], with no data currently available for the 10-5 RJT. The present study is the first to examine the reliability of RSI in the 10-5 RJT through the use of the Output Capture IMU; so this should also be examined further in future studies to challenge or accept the current findings. Future research should also analyse the change in TE using the Output IMU within different population groups. Furthermore, future research protocols should look at the effect of additional familiarisation sessions for the DJ and 10-5 RJT, as these may result in an overall reduction in the TE of both tests.

Due to the lack of published studies on the Output Sports IMU, future research protocols must examine the validity of the IMU in measuring RSI further. The current study has determined that the Output Capture IMU is reliable in measuring RSI in both the DJ and 10-5 RJT and has challenged the reliability findings of the previous literature; however, it does not question the validity findings of previous studies [22]. The results of the current study do not reflect whether it is a reliably accurate or inaccurate reflection of RSI. Determining the validity of the IMU is imperative to practitioners as it will ascertain whether the device demonstrates any element of systematic bias in RSI and its component measures of JH and GCT.

## 5. Conclusions

In conclusion, the Output Capture IMU demonstrated that it was reliable in the measurement of RSI derived from the DJ and 10-5 RJT. The results of this study cast doubt as to the usefulness of the IMU in detecting the SWC of RSI in the same tests; however, it is able of detecting a moderate change. The results provide practitioners with the knowledge that the Output Capture IMU is reliable for practical use when monitoring RSI in fast-SSC plyometric activity.

Given that the Output Capture unit is a relatively new device to the market with limited research, the results of this study will provide practitioners with some indicative data on RSI reliability, which can be used when determining what device may be of use to them in the monitoring of data with field-based athletes. As the TE was greater than the SWC (0.2) in both the DJ and 10-5 RJT, this may indicate that there may have been variance within individuals in the participant cohort. As such, practitioners using the Output Capture device should avoid the use of the SWC (0.2) to determine small meaningful RSI change in acute monitoring or should go through extended familiarisation periods with athletes prior to testing. The tests can be used however to detect the SWC (0.5) for moderate meaningful change, as the TE was less than the SWC (0.5) in both the DJ and 10-5 RJT.

Furthermore, when practitioners are testing for RSI, the same test for determining RSI should be used between different time points. The test that is conducted should be the same; therefore, the DJ and 10-5 RJT should not be used interchangeably. Similarly, a practitioner should not employ the use of separate devices interchangeably to measure RSI in athletes over time. Concurrent validity and reliability research of RSI in currently available devices that measure RSI states that practitioners should use only one device when testing over time to allow for a higher level of reliability within- and between-subject variance.

In practical application, the Output Capture IMU may be used for longitudinal monitoring of RSI in field-based athletes where the aim is to track moderate changes in fast-SSC performance rather than small acute fluctuations. Practitioners should ensure that testing protocols, warm-up procedures, jump techniques, and consistent placement of an IMU are standardised across sessions when using the device in order to maximise the interpretability of RSI data over time.

## Figures and Tables

**Table 1:** Average reactive strength index (RSI) across three trials per participant for drop jump (DJ) and 10-5 rebound jump test (10-5 RJT), mean ± standard deviation (SD).

Participants	Trial 1	Trial 2	Trial 3
DJ	1.64 ± 0.26	1.65 ± 0.26	1.69 ± 0.30
10-5 RJT	1.59 ± 0.24	1.60 ± 0.21	1.56 ± 0.23

**Table 2:** Reliability and usefulness of the RSI as derived from the DJ and 10-5 RJT. 95% CI—95% Confidence Interval; ICC—Intraclass Correlation Coefficient; CV—Coefficient of Variation; TE—Typical Error; SWC—Smallest Worthwhile Change.

		95% CI			95% CI						
Variable	ICC	Lower	Higher	CV%	Lower	Higher	TE	SWC 0.2	Rating	SWC 0.5	Rating
DJ	0.89	0.78	0.95	6.2	5.0	8.2	0.09	0.05	Marginal	0.14	Good
10-5 RJT	0.89	0.77	0.95	5.2	4.2	7.2	0.08	0.04	Marginal	0.11	Good

## Data Availability

The data that support the findings of this study are available from the corresponding author upon reasonable request.

## Abbreviations

The following abbreviations are used in this manuscript:

SSC	Stretch-shortening cycle
GCT	Ground contact time
RSI	Reactive strength index
JH	Jump height
DJ	Drop Jump
10-5 RJT	10-5 rebound jump test
CV	Coefficient of variation
ICC	Intraclass correlation coefficient
SWC	Smallest worthwhile change
IMU	Inertial measurement unit
PAR-Q	Physical activity readiness questionnaire
TE	Typical error
